# Effect of vaginal douching on vaginal flora and genital infection

**DOI:** 10.4274/jtgga.galenos.2019.2018.0133

**Published:** 2020-03-06

**Authors:** Rasime Yıldırım, Gülşen Vural, Esra Koçoğlu

**Affiliations:** 1 Department of Health Care Services, Kastamonu University Tosya Vocational High School, Kastamonu, Turkey; 2 Department of Obstetrics and Gynecology, Near East University Faculty of Nursing, Nicosia, Cyprus; 3 Department of Medical Microbiology, İstanbul Medeniyet University Faculty of Medicine, İstanbul, Turkey

**Keywords:** Vaginal douche, vaginal flora, infection

## Abstract

**Objective:**

This study aimed at examining the effect of vaginal douching (VD), which is a traditional and cultural application, on the vaginal flora and genital infections.

**Material and Methods:**

This descriptive study included 190 women including those who did or did not perform VD. A questionnaire survey and vaginal sampling were employed. The collected samples were transported within 8 h for laboratory testing.

**Results:**

There was no significant difference between the two groups in terms of vaginal flora. In the VD group, only a few patients reported a history of Sexually Transmitted disease (STD), but none in the non-VD group had STDs (p<0.05). No significant difference in infections was noted. However, there was a significant relationship between the history of infections and VD (p<0.01).

**Conclusion:**

Women who performed VD are at risk for vaginal infections. Further studies are warranted in the future for clinical application.

## Introduction

Vaginal douching (VD) is the process of washing the vagina with water or other liquid solutions (1,2). VD can be widely seen in cultures that define the female body, menstruation, and sexual relations as dirtiness. In Turkish culture, women define menstruation as dirtiness (3). In Turkey, the rate of VD was 43.9-64.5% (2,4,5,6,7,8,9). In a 2014 study by the Republic of Turkey Ministry of Health Department, 79.20% women were found to be douching for hygiene (96.26%), religious belief (52.86%), and pregnancy prevention (12.74%) (10). These women stated that douching helped them feel clean, healthy, and good, treated infections, provided ablution, enhanced their appeal to partners, and prevented pregnancy. Moreover, women performed VD for vaginal cleaning following coitus to protect themselves from diseases, during menstruation, to feel clean before sexual intercourse and gynecologic examinations, to decrease unpleasant odours, to imitate others who performed VD, to gain experience, or out of curiosity (2,4,5,6,8,9,11,12,13,14).

Various researchers have evaluated the effects of VD on the health of women. Although some studies emphasised that VD caused important health issues, others revealed no such correlation. Some studies have indicated an effect of VD on vaginal flora and on the ascension of microorganisms into the upper genital tract (15,16). In the past, VD was associated with bacterial vaginosis, human immunodeficiency virus (HIV), and chlamydial infections, pelvic inflammatory disease (PID), preterm birth, low-birth-weight infants, infertility, ectopic pregnancy, cervical cancer, and AIDS (14,17,18,19). Vaginal dryness, burning in the vaginal area, genitourinary infection, and irritation have been reported in women who douche frequently (18). In a past study, the rates of genital infections were 53.5% and 33.8% in women who did and did not douche, respectively (19). In 1990, Brinton et al. (20) found that the risk of cervical cancer and PID increased with the use of commercial products instead of water and soap solutions during douching. In 2006, Akın et al. (6) detected the rate of VD in women with a history of infertility (40.0%), miscarriage (47.3%), preterm birth (40.0%), and low-birth-weight infants (57.1%) (p>0.05). These results indicate the variation in the reported findings on VD. Martino and Vermund (16) emphasised that VD was harmful. The World Health Organization has also indicated the adverse effects of VD in 2012. In a study by Sunay et al. (22), women who douched demonstrated an increased risk of abnormal vaginal discharge (about 3.9 times more; p=0.001) than women who did not douche.

Some studies support the positive effect of douching on health. For instance, some studies reported the alleviating effect of VD on HIV and human papilloma virus. In fact, antiseptic douche solutions have been shown to decrease the incidence of HIV (23,24). In a study on the effect of douching on vaginal flora, douching with saline or acetic acid once daily was found to reduce the structure and number of vaginal bacteria within 10 min. Moreover, douching with povidone-iodine-like bactericidal agents reportedly induced over-reproduction of pathogenic organisms that repress *Lactobacillus* (25,26). Hence, this study aimed to detect the effect of VD on vaginal flora and genital infection in women aged >18 years.

## Material and Methods

This study was conducted at the Ministry of Health Hospital and the Maternal and Infant Health Centre of Family Planning. Study subjects included women who had been referred to these centres. The sample size was calculated using the NCSS Pass 2008 program, which required 190 women. Sen and Mete (2) reported a VD frequency of 47.2%. The ratio of VD was predicted as 27.2-67.2% using 95% confidence interval (CI) values, 80% power, and 20% standard deviation. On the basis of their douching behaviour, subjects were divided into two study groups: douching and non-douching groups. The VD group consisted of women who had douched in the last 3 days because the effects of douching on vaginal flora continue for 3 days.

The sample selection criteria were age ≥18 years; not being pregnant; no delivery date in the first 42 days; non-diabetic; not in their menstrual cycle during the study period; not using immunosuppressive drugs, antibiotic, antifungal, antiviral, corticosteroid or chemotherapy use in the past 2 weeks and no sexual intercourse in the past 3 days.

A questionnaire developed by the researchers based on the literature was used for data collection. Written informed consent was obtained from the subjects before data collection. After administering the questionnaire, vaginal samples were collected by the researcher in a private room. Vaginal samples were taken from the posterior wall of the vagina and lateral fornix without contacting the vulva using a sterile and disposable cotton swab.

A single-blind study was conducted for the cultivation and examination of vaginal samples by a microbiologist. The samples were transported in Stuart Transport Medium to the Laboratory of Bolu Abant İzzet Baysal University Health Research and the Application Center Microbiology Laboratory within 8 hours of sample collection. Before analysis, the samples were stored at room temperature (transport medium is stable at room temperature). Direct examination and cultivation were performed under laboratory conditions.

### Ethical aspect of the study

Ethical permissions were obtained from the Governorship of Bolu city; Bolu Abant İzzet Baysal University, Health Research and Application Center, Microbiology Laboratory; Bolu Abant İzzet Baysal University Faculty of Medicine, Clinical Research Ethical Committee (decision no: 2011/26). Before applying questionnaire, written consent was taken from the women by giving information about the study. The expenses of the laboratory and stationery equipment were met by the financial support of Selçuk University, Coordination Office of Scientific Research Projects.

### Statistical analysis

The SPSS 15.0 program was used for the statistical analysis of the study data. Study data were evaluated using the chi-square and logistic regression tests. A p value <0.05 was considered statistically significant.

The presence of vaginal infection pathogens and women’s health-related factors were considered dependent variables. Factors including age, educational background, working status, health insurance, and the level of income were the independent variables.

## Results

The rate of douching was much higher in women aged ≥50 years (61.1%). All women in the non-VD group were in the 20-29 years age group (57.4%), and they were all secondary school graduates. The educational level was higher for women in the non-VD group (76.6%). Among the employed women, 61.1% reported not douching, and 56.8% of unemployed women reported douching. The income range of douching women was 120-320 United States Dollar (USD) (59.6%) and <120 USD (59.3%), whereas that of women in the non-VD group was ≥500 USD (80.0%) and 320-500 USD (61.2%).

Of the total, 41.1% women reported having heard about VD from their social group, 37.9% decided to douche by themselves, and 9.5% learnt it from their mothers. The frequency of douching was 3-4 times/week by 35.8% and 1-2 times/week by 33.7% of the women. The frequency of douching was the highest after sexual intercourse in 69.5% of women.

Most women performed VD for personal hygiene. The reasons for douching were reported to be personal hygiene by 83.2%, religious reasons by 26.3%, protection from diseases by 9.5%, family planning by 5.3%, and ignorance by 2.1%. Moreover, 75.8% of women douched with water, whereas 17.9% douched with soap.

A statistically significant difference was found regarding vaginal infection history (p<0.01) between the two groups. In the VD group, 2.1% of women had Sexually Transmitted disease (STD) previously, whereas no women in the non-VD group had STD. Hepatitis B was reported in two women. The previous incidence of vaginal infection was 57.9% in VD subjects and 37.9% in non-VD subjects. A statistically significant difference was found between women with vaginal infection and those performing VD (p<0.01). In women with vaginal infection, the reason for infection was unknown in 78.7% in the VD group and 63.9% in the non-VD group.

No statistically significant difference was found between the two groups with respect to vaginal flora (p>0.05). The microbiologic evaluation results of vaginal flora revealed that the rate of women with normal vaginal flora (the primary colonising bacteria of a healthy individual is Lactobacillus) was 57.9% in the VD group and 70.5% in the non-VD group (p>0.05) (Table 1).

According to the result of logistic regression, a statistically significant difference was determined between the working status, profession, education level, and income status of the women (p<0.01) (Table 2). The probability of VD was found to be higher in housewives and workers in comparison with that in employed women and civil servants [odds ratio (OR)=2.064, 95% confidence interval (CI): (1.136-3.753); OR=4.185, 95% CI: (1.520-11.521), respectively]. When the education levels were similarly investigated, the probability of VD was found to be much higher in women had or had not graduated from primary school [OR=4.052, 95% CI: (1.669-9.837)] and who had graduated from middle school (OR=5.564, 95% CI: 1.981-15.623) and high school [OR=4.792, 95% CI: (2.092-10.976)] in comparison with that in university graduates. Women with low income were likely to douche more often than women with high income [OR=5.895, 95% CI: (1.829-19.003)].

In the logistic regression analysis (reference, douching; risk factor, non-douching), the incidence of genital infection was higher in the VD group than in the non-VD group [OR=2.253, 95% CI: (1.260-4.029)].

## Discussion

In the VD group, 59.4% of women had primary school and lower education, and 63.0% had graduated from middle school. The education level in the non-VD group was determined to be high school in 44.7% and higher education in 76.6% of women (p<0.01); these findings are supported by other studies (2,4,5,6,7,16,19). On the basis of these results, a reverse correlation exists between the education level and VD frequency.

Among the employed women, 61.1% reported not performing VD, whereas 56.8% of the unemployed women reported performing douching (p<0.05). In studies performed by Karaer et al. (5) and Ege et al. (19), employed women were less likely to perform VD. Another study, Yanikkerem and Yasayan (11) reported that 81.9% of the women who performed VD were housewives. The similarity between this study and other studies was revealed in terms of the working status of the women. A relationship was reported between VD and the socioeconomic levels of women. In the present study, the incidence of VD was higher in women with low income (p<0.01). Karaer et al. (5) also reported a statistically significant relationship between the level of income and VD, as in our study (p<0.01). On the contrary, Sunay et al. (22) found that the frequency of performing VD was higher in married and low-income women.

Women had learnt VD from their social groups (41.1%), by themselves (37.9%), through their mothers (9.5%), through healthcare personnel (9.5%) and through the media (2.1%). Thus, the sources of learning VD were elders, media, mothers, family members, their friends, healthcare personnel, and relatives (2,4,6,7,8,11,12,14,27). A study by Rosenberg et al. (28) showed that douching had a strong cultural component. The frequency of VD was 35.8% for 3-4 days/week and 33.7% for 1-2 days/week. Overall, the frequency of VD ranges between 1 and 2 times per day and between 1 and 2 times per week/month (4,6,7,8,9,11,13,29). The factors affecting the frequency of VD were determined as their cause, practice time, belief, and cultural response. It was believed that the high incidence of VD was related to avoiding infection from the toilet, menstruation, and sexual intercourse.

Most women used water (75.8%) and a solution of soapy water (17.9%) during VD. Various materials were identified for douching, primarily with water, and the second most commonly used product was water with soap (4,7,8,9,11,19). Similar studies performed in other countries revealed that water containing vinegar and commercial solutions were used more frequently for VD (1,13). With respect to the need for maintaining personal hygiene, it may be thought that VD was performed using only water or a solution of soapy water after taking a bath and using the toilet without the use of other solutions. Moreover, the incidence of VD was higher among women with low socioeconomic status. Therefore, the reason for using a solution of soapy water was considered related to their low costs.

The effects of these VD solutions on the vaginal flora remain unknown. In 2004, Zhang et al. (30) identified the incidence of bacterial vaginosis in women using water with vinegar for VD. In 1992, Onderdonk et al. (25) found that povidone-iodine caused a significant reduction in the normal flora (lactobacilli, the dominant bacteria in the vagina) and an increase in the incidence of vaginal infections. In 2000, Pavlova and Tao (31) reported that the inhibitory effect of solutions containing vinegar on pathogens caused vaginal infections, except on Lactobacillus. In this study, when the effects of solutions used for VD were investigated, the rate of using a water and soap solution was 14.5% in women with a normal vaginal flora and 22.5% in women with vaginal infection in the VD group. Moreover, 80% of the women with a normal vaginal flora used water and 70% of women with vaginal infection also used water.

In this study, the frequency of using water and soap solution was higher in women with vaginal infection than in women with normal vaginal flora. The rate of using water alone by women with normal vaginal flora was higher than that by women with vaginal infections. According to these results, the rate of using water alone for douching was lower than that of using water and soap solution. Van Royen et al. (32) determined that women with bacterial vaginosis used greater amounts of soap for hygiene purposes. However, it remains unknown whether the use of soap causes any change in the vaginal flora and the reason for frequent bathing may be the presence of a fishy odour in the vaginal discharge.

In this study, 57.9% of women in the VD group had an infection history, whereas 62.1% of women in the non-VD group had no infection history (p<0.01). Similar results were obtained by other studies; for instance, the rates of vaginal infections were higher in VD groups than in non-VD group (4,6,7,9,11,19). Although these findings support that VD may be a risk factor for vaginal infections, the frequency of VD was 1-2 days/week in 40% of the women with vaginal infection compared with 3-4 days/week in those with normal vaginal flora. Women evaluated in terms of the rate of performing VD did not have any effect on the change in flora.

The incidence of having normal vaginal flora was 57.9% in the VD group and 70.5% in the non-VD group (p>0.05) (Table 1). When the reasons for douching and factors affecting these reasons were evaluated, profession, education, and income levels were statistically significant (p<0.01). The probability of VD was higher in housewives and unemployed women than in employed women and civil servants [OR=2.064, 95% CI: (1.136-3.753)]; OR=4.185, 95% CI: (1.520-11.521), respectively). Similarly, when the education levels were investigated, the probability of VD was much higher in women who did or did not graduate from primary school [OR=4.052, 95% CI: (1.669-9.837)] and those who graduated from middle school [OR=5.564, 95% CI: (1.981-15.623)] and high school [OR=4.792, 95% CI: (2.092-10.976)] than those in graduates. Women with low income were likely to douche more frequently than women with high income (Table 2). Sen and Mete (2) and Arslantas et al. (8) reported that education level was statistically significant when VD-related parameters were evaluated through logistic regression analysis (p<0.009 and p<0.001, respectively). The former found that the probability of VD was higher among illiterate women than in educated women [OR=1.760, 95% CI: (1.154-2.683)]. On the contrary, the latter found that the probability of VD was lower in women with a college or university degree [OR=0.02, 95% CI: (0.005-0.09)]. According to Arslantas et al. (8), a statistically significant relationship was evident between VD and working status (p=0.004), and the probability of VD was lower among employed women [OR=0.34, 95% CI: (0.16-0.70)]. The findings of our study, which are similar to those of Sen and Mete (2) and Arslantas et al. (8), imply that the probability of VD was reduced as a result of increasing education level and working outside of the home.

In the present study, logistic regression analysis was performed to determine the effect of VD on genital infection history. The incidence of genital infections was higher in the VD group than in the non-VD group [OR=2.253, 95% CI: (1.260-4.029)]. Therefore, VD may predispose women to vaginal infections. Sunay et al. (22) reported that the risk of vaginal discharge was 3.9 times higher in the VD group than in the non-VD group [p=0.001; OR=3.86, 95% CI: (0.651-1.534)]. Consequently, there was no statistically significant difference in terms of infection as a result of microbiologic evaluation of vaginal samples. However, a statistically significant relationship was determined between infection history and VD (p<0.01). Therefore, we believe that women who perform VD are at risk for vaginal infections. Further studies are recommended to understand this issue better.

Some of the participating women who presented to the study centres met the sample exclusion criteria.

## Figures and Tables

**Table 1:**
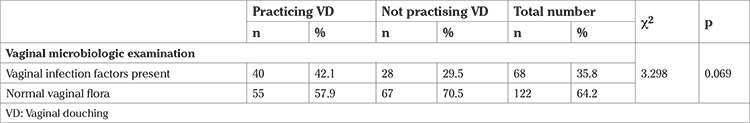
Distribution of female vaginal specimens according to microbiologic examinations (infection effect-normal flora presence) according to vaginal douching status (n=190)

**Table 2:**
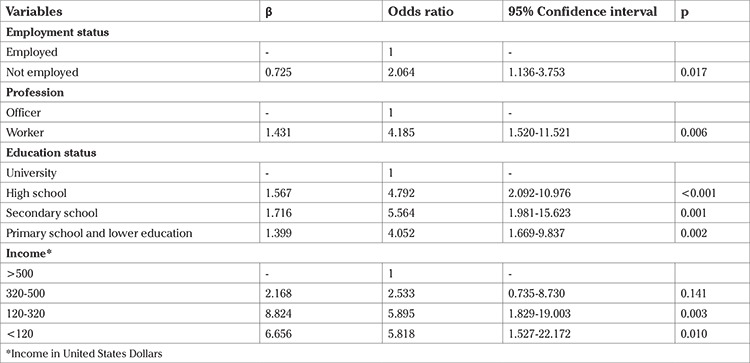
Investigation of some variables affecting women’s vaginal douching behaviours using logistic regression
